# Primary Synovial Osteochondromatosis of the Elbow in a Young Adult Complicated by Osteoarthritis: A Case Report

**DOI:** 10.7759/cureus.85805

**Published:** 2025-06-11

**Authors:** Abdoulrazak Egueh Nour, Chirwa Abdillahi Mahamoud, Chaimae Amrani, Bouknani Nawal, Amal Rami

**Affiliations:** 1 Radiology, Cheikh Khalifa International University Hospital, Casablanca, MAR

**Keywords:** arthroscopic synovectomy, elbow joint, loose bodies, secondary osteoarthritis, synovial osteochondromatosis

## Abstract

Primary synovial osteochondromatosis is a benign and uncommon pathology, resulting from metaplasia of the synovial tissue, of unknown cause, leading to the formation of multiple cartilaginous nodules, some of which secondarily detach to form free articular bodies. Its location in the elbow, complicated by osteoarthritis, is poorly described in the literature. We present the case of a 25-year-old man presenting for chronic right elbow pain with joint locking and swelling without a history of trauma. Physical examination revealed an increase in elbow volume compared to the contralateral elbow, with limitation of active or passive joint range of motion. Radiological examinations, including standard radiography (X-rays), CT, and MRI, revealed multiple rounded, floating, and calcified bodies disseminated in the joint associated with arthritic changes, confirming the diagnosis of primary synovial osteochondromatosis complicated by osteoarthritis. Synovectomy combined with arthroscopic debridement and removal of loose bodies was performed, resulting in significant improvement in pain and joint function. Pathological examination confirmed the diagnosis of primary synovial osteochondromatosis. Elbow involvement is unusual and often diagnosed late due to nonspecific symptoms. Imaging is essential for early diagnosis and to assess synovial extension and cartilaginous complications.

## Introduction

Primary synovial osteochondromatosis is a benign and uncommon pathology, characterized by cartilaginous metaplasia of the synovial membrane, leading to the formation of osteochondral nodules within the joint cavity [1]. The disease is classically monoarticular and mainly affects the large joints, particularly the knee, while elbow involvement is less common [2]. Due to the rarity of this localization, its true prevalence is difficult to establish. The majority of published data consists of isolated case reports or small case series, with the largest series reporting up to 30 cases [3]. But any structure covered with synovial fluid, including all synovial joints, may be affected.

Occurring most often between the ages of 30 and 50 and more widespread in men, this pathology has a slow and progressive evolution and manifests itself by joint pain, swelling, functional discomfort, or blockages [4]. Multimodal imaging, including X-ray, CT scan, and MRI, plays a vital role in early diagnosis as well as assessment of synovial and articular cartilage involvement.

Treatment is based on surgical management, combining synovectomy and removal of loose bodies, while limiting the risk of recurrence or arthritic progression [5].

## Case presentation

A 25-year-old manual worker with no significant medical or traumatic history presented with mechanical pain in his right elbow, which had been developing for three months and had increasing functional impairment. He also reported episodes of locking and progressive limitation of joint range of motion.

Clinical examination revealed moderate joint effusion, pain on palpation of the olecranon fossa and humeroradial joint space, and limited active extension and flexion. There were no local inflammatory signs or associated neurological or vascular involvement.

The biological workup, including complete blood count, C-reactive protein (CRP), erythrocyte sedimentation rate (ESR), and rheumatoid factor, was within normal limits, thereby excluding an underlying inflammatory or autoimmune disorder, as seen in Table 1.

**Table 1:** Blood testing results TSH: thyroid-stimulating hormone; ESR: erythrocyte sedimentation rate.

Red blood cells (10^12^ / L)	5,35	4.25-6
Hemoglobin (g/dL)	15,200	13.0-18.0
Hematocrit (%)	44,600	39-53
Leukocytes (10^3^/mm³)	6,800	4-11
Platelets (10^3^/mm³)	240	150-400
Blood creatinine (mg/L)	8,700	6.7-11.7
Protein Totals (g/L)	68	64-83
Calcium (mg/L)	93	86-100
Urea (g/L)	0,23	0.17-0.49
Triglycerides (g/L)	0,89	<1.30
Glycosylated Hemoglobin (HbA1c) (%)	5,300	4.5-6.2
Serum Ferritin (ng/ml)	251	30-400
TSH us (µIU/mL)	0,71	0.27-4.20
ESR 1st hour (mm/h)	10	<13
C-reactive Protein (mg/L)	5	< 8
Rheumatoid Factors (IU/ml)	<14	<30

**Figure 1:**
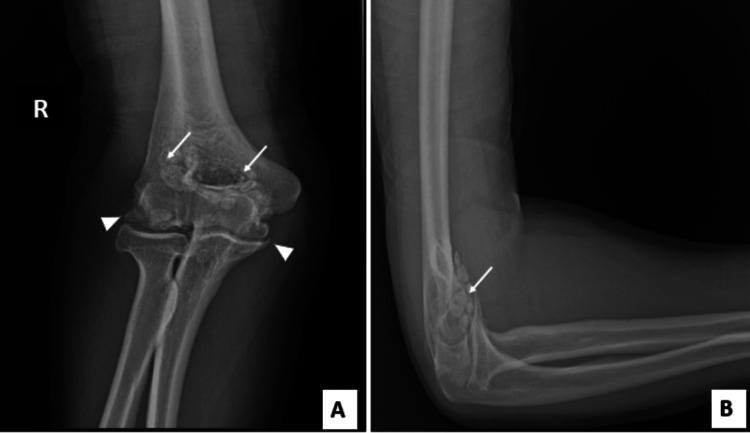
X-rays of the right elbow (A) anteroposterior view (B) lateral view Revealed multiple intra-articular loose bodies (arrow), pinching of the joint space, subchondral sclerosis, and osteophytes (arrowhead).

**Figure 2:**
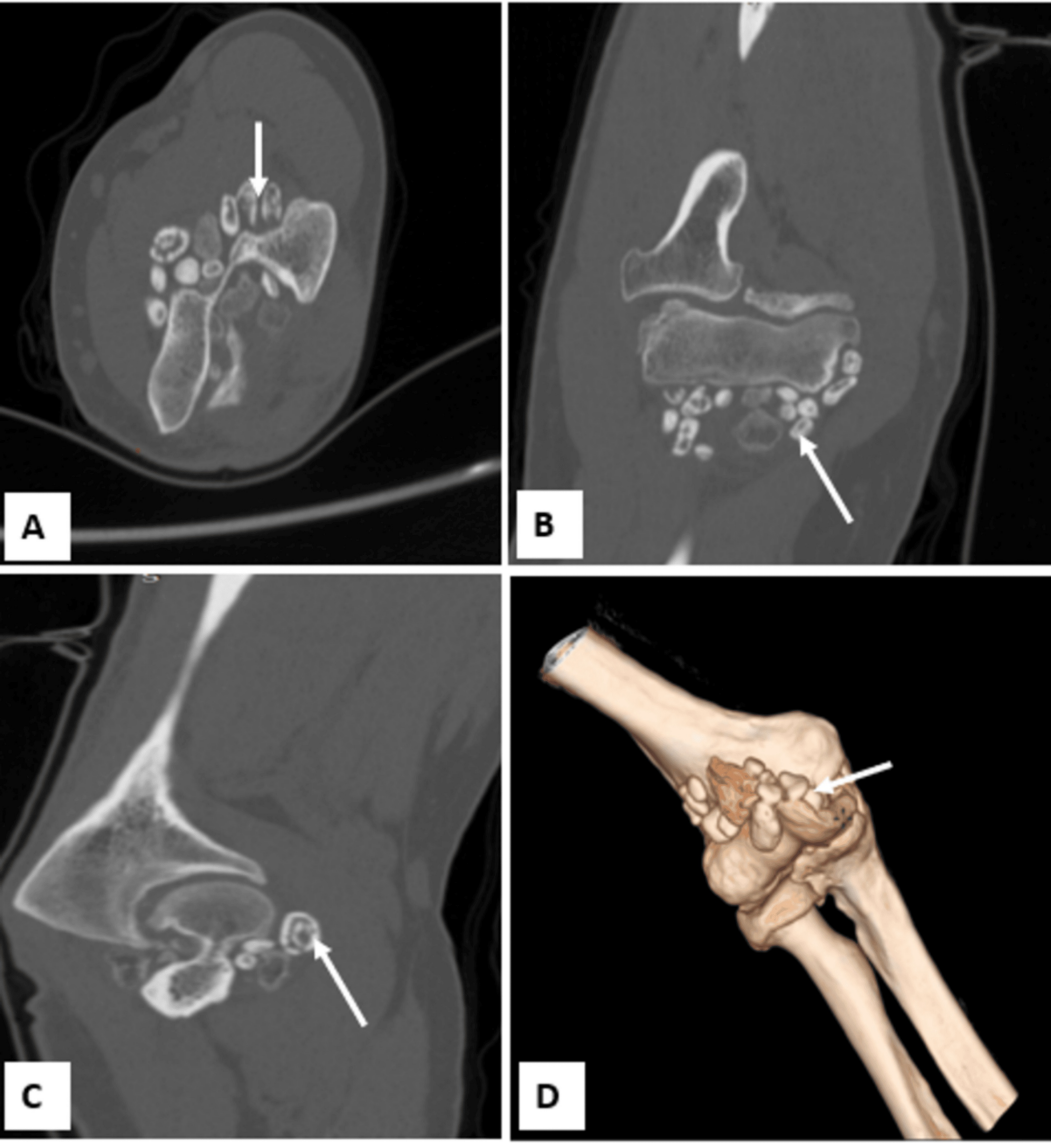
CT scan of the elbow (A) axial plane, (B) coronal plane, (C) sagittal plane, (D) three-dimensional reconstruction Showed multiple calcifications of the periarticular soft tissues(white arrow), associated with sclerotic lesions and pinching of the joint space.

Standard radiography of the elbow revealed multiple rounded intra-articular opacities, suggesting free osteocartilaginous bodies associated with joint narrowing, subchondral changes, and osteophyte formation (Figure 1). Non-contrast CT showed grouped intra-articular calcifications in the olecranon fossa, in the coronoid fossa, confirming their presence in the anterior and posterior compartments (Figure 2).

**Figure 3:**
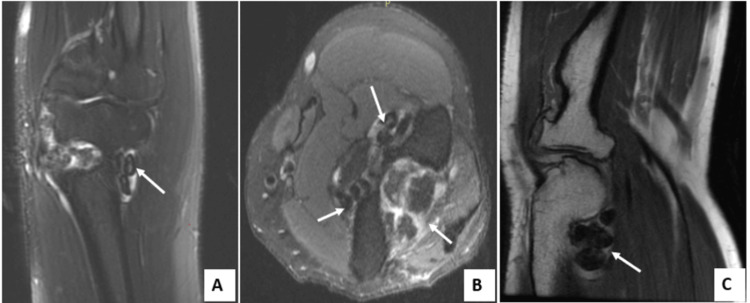
T2-weighted MRI (A) coronal, (B) axial; T1-weighted MRI (C) sagittal Shows joint effusion and multiple hypointense nodules on T1- and T2-weighted sequences, consistent with cartilaginous loose bodies, as well as associated degenerative changes and synovial thickening.

Magnetic resonance imaging showed thickened synovium, intra-articular effusion, as well as several intra-articular nodular formations in T1 and T2 hypointense, reflecting osteochondral bodies and cartilaginous lesions compatible with early osteoarthritis (Figure 3).

**Figure 4:**
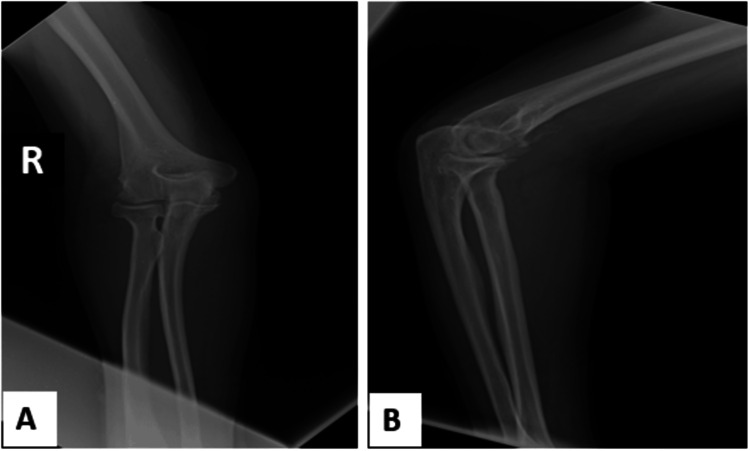
Postoperative radiographs of the right elbow (A) anteroposterior (B) lateral Shows no calcific densities.

The diagnosis of primary synovial osteochondromatosis, complicated by osteoarthritis, was made. Surgical synovectomy with removal of loose bodies was performed. Histopathological analysis confirmed the diagnosis of primary synovial osteochondromatosis without signs of malignancy. The post-operative outcome was favorable, with functional rehabilitation and a clear improvement in pain and joint mobility at follow-up. At six months, radiographic control showed no residual calcified bodies, indicating effective complete excision (Figure 4).

## Discussion

Synovial osteochondromatosis (SOC) is an uncommon benign condition related to synovial metaplasia leading to the formation of multiple intra-articular cartilaginous nodules [1].

It can be primitive of idiopathic origin or secondary to an underlying joint pathology, such as osteoarthritis, trauma, inflammatory arthropathies, or neurological damage,e and can be both intraarticular and extraarticular [4,6].

Described in the 16th century by Ambroise Paré, then studied by Laennec and Brodie, joint damage by free cartilaginous bodies has historically been associated with a synovial origin [7]. The first case reported at the elbow dates back to 1918, but the disease can affect any synovial joint.

The disease is generally monoarticular and preferentially affects men between 30 and 50 years of age with a clear predominance in the knee; locations in the elbow, hip, shoulder or ankle are less frequent and manifest clinically by chronic pain, swelling, stiffness and sometimes mechanical blockage linked to the presence of free bodies [8,9].

In terms of evolution, Milgram and Pease established, from a series of 30 cases, a three-stage classification of synovial osteochondromatosis, based on histopathological criteria, ranging from active synovitis without free bodies (stage I) to a form with ossified free bodies (stage II) responsible for mechanical symptoms and inactive synovitis with persistence of ossified free bodies (stage III) [3]. Our patient falls into this latter stage. Edeiken et al. then proposed a stage IV, characterized by a large intra- or extra-articular cartilaginous mass [10].

Imaging plays a central role in the diagnosis and characterization of primary synovial osteochondromatosis. Standard radiography is the first-line examination and can reveal rounded, calcified opacities corresponding to intra-articular osteochondral bodies. However, in early non-calcified forms, the results can be falsely reassuring. In this context, computed tomography or MRI is useful to confirm the diagnosis. Computed tomography (CT) allows for more precise mapping of loose bodies and associated bone remodeling, particularly in cases of secondary osteoarthritis. More sensitive magnetic resonance imaging (MRI) completes the evaluation by visualizing soft tissues, hypertrophied synovium, effusions, as well as possible cartilaginous or extra-articular involvement, and non-calcified forms [9].

No biological marker is specific to the primary form, but certain anomalies can point towards a secondary form linked to an underlying joint pathology [11].

The differential diagnosis of synovial osteochondromatosis includes degenerative, inflammatory conditions (such as rheumatoid arthritis or gout, benign tumors such as periosteal chondroma or giant cell tumor, pigmented villonodular synovitis, and hydroxyapatite deposits. Finally, malignant lesions such as chondrosarcoma or synovial sarcoma must be systematically excluded, particularly in recurrent or atypical forms [9]. Cross-analysis of imaging, clinical, and biological data often allows for a reliable diagnosis.

The standard treatment for synovial osteochondromatosis is the excision of loose bodies, often by arthroscopic route, associated or not with a synovectomy [12]. In our case, a synovectomy of the anterior and posterior compartments was performed. The prognosis is generally favorable, but recurrences are possible, often linked to incomplete excision, and malignant transformation into chondrosarcoma, although rare, has been described, mainly in recurrent forms.

## Conclusions

Primary synovial osteochondromatosis, although unusual in the elbow, should be included in the differential diagnosis in the presence of chronic pain, joint blockages, and loose bodies visible on imaging. Multimodal evaluation by radiography, CT, and MRI is essential for an accurate and early diagnosis. Surgical excision combined with synovectomy offers good functional results and limits the risk of recurrence. Finally, rigorous clinical and radiological monitoring is essential for early detection of any complications.
